# No socioeconomic inequalities in colorectal cancer survival within a randomised clinical trial

**DOI:** 10.1038/sj.bjc.6604743

**Published:** 2008-10-28

**Authors:** U Nur, B Rachet, M K B Parmar, M R Sydes, N Cooper, C Lepage, J M A Northover, R James, M P Coleman

**Affiliations:** 1 Cancer Research UK Cancer Survival Group, Non-Communicable Disease Epidemiology Unit, London School of Hygiene and Tropical Medicine, Keppel St, London WC1E 7HT, UK; 2 Cancer Group, MRC Clinical Trials Unit, 222 Euston Road, London NW1 2DA, UK; 3 National Cancer Intelligence Centre, Social and Health Analysis and Reporting Division, Office for National Statistics, (Room FG/114) 1 Myddelton Street, London EC1R 1UW, UK; 4 Registre bourguignon des cancers digestifs [INSERM EMI 0106], Faculté de Médecine, BP 87900, Dijon Cédex F-21079, France; 5 St Marks Hospital, Northwick Park, Watford Road, Harrow, Middlesex HA1 3UJ, UK; 6 Kent and Medway Cancer Network, Preston Hall, London Rd, Aylesford, Kent ME20 7NJ, UK

**Keywords:** colorectal cancer, rectum cancer, cancer survival, deprivation, cancer registries

## Abstract

There is strong evidence that colorectal cancer survival differs between socioeconomic groups. We analysed data on 2481 patients diagnosed during 1989–1997 and recruited to a randomised controlled clinical trial (AXIS, ISRCTN32414363) of chemotherapy and radiotherapy for colorectal cancer. Crude and relative survival at 1 and 5 years was estimated in five categories of socioeconomic deprivation. Multiple imputation was used to account for missing data on tumour stage. A multivariable fractional polynomial model was fitted to estimate the excess hazard of death in each deprivation category, adjusting for the confounding effects of age, stage, cancer site (colon, rectum) and sex, using generalised linear models. Relative survival in the trial patients was higher than in the general population of England and Wales. The socioeconomic gradient in survival was much smaller than that seen for colorectal cancer patients in the general population, both at 1 year −3.2% (95% CI −7.3 to 1.0%, *P*=0.14) and at 5 years −1.7% (95% CI −8.3 to 4.9%, *P*=0.61). Given equal treatment, colorectal cancer survival in England and Wales does not appear to depend on socioeconomic status, suggesting that the socioeconomic gradient in survival in the general population could well be due to health-care system factors.

Cancer survival differs between socioeconomic groups (Kogevinas and Porta, 1997; Woods *et al*, 2006). This has been demonstrated for many adult cancers, including those of colon and rectum, both in England and Wales (Coleman *et al*, 1999, 2001, 2004) and in Scotland (Stockton, 2002; Shack *et al*, 2007). The origin of these inequalities in survival remains largely unexplained and controversial. Although late stage of disease at diagnosis is likely to explain in part the lower survival among patients living in deprived areas (Woods *et al*, 2006), in particular for colorectal cancer (Mitry and Rachet, 2006), most recent research suggests that other factors play an important role, such as differential access to treatment or differential disease management by the health-care system (Mitry and Rachet, 2006; Woods *et al*, 2006).

Clinical trials enable us to test the hypothesis that differential treatment underpins the socioeconomic survival gradients in the general population, because patients are randomly allocated into treatment groups and all patients theoretically receive the same treatment, with close adherence to protocol, regardless of their socioeconomic status.

Our objective was to quantify socioeconomic differences in survival among patients recruited to a randomised controlled trial. We reasoned that if the socioeconomic gradient in survival was abolished in the setting of a trial, with equal treatment for all patients, then differences in treatment would become a more plausible explanation for the socioeconomic differences in survival as seen in the general population.

## Materials and methods

**Figure 1:**
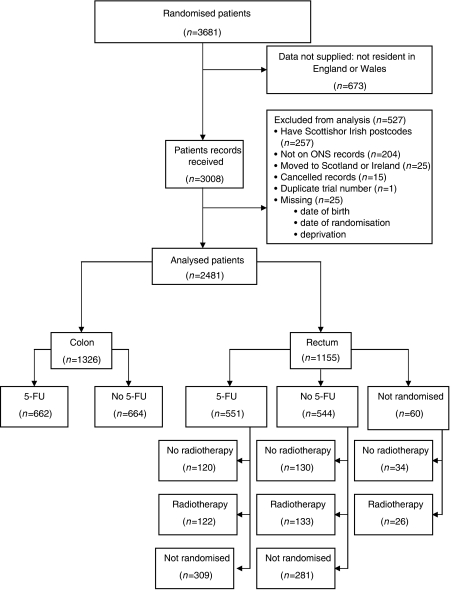
Distribution of patients by cancer site (colon, rectum) and randomisation to postoperative portal vein infusion (PVI) of 5-fluorouracil (5-FU) or radiotherapy (RT).

The Medical Research Council (MRC) Clinical Trials Unit (CTU) carried out a randomised clinical trial of adjuvant radiotherapy and 5-fluorouracil (5-FU – a chemotherapy agent) infusion for patients with colorectal cancer (AXIS trial, ISRCTN32414363). All colon cancer patients and all but 60 of the rectal cancer patients were randomised before or during surgery to postoperative portal vein infusion (PVI) of 5-FU, or else no PVI. Half (49%) of the rectal cancer patients were also randomised to radiotherapy or no radiotherapy in a partial factorial design (Figure 1). A detailed description has been published. No evidence of benefit was found, either for PVI or for radiotherapy (The AXIS collaborators, 2003).

The AXIS trial began before the current system of Multi-Centre Research Ethics Committees (MREC), although each participating site had the appropriate local ethical approval. No REC was prepared to evaluate the proposal to exploit the data from this trial for a different purpose after its closure, so approval was obtained from the ethics committee of the London School of Hygiene and Tropical Medicine and from the independent AXIS Trial Steering Committee, with support from the Chief Investigator.

The AXIS trial was designed to test whether 5-FU or radiotherapy would improve overall and disease-free survival in patients with resectable primary malignancy of the bowel. A total of 3681 patients met the initial eligibility criteria, namely the presence of suspected colorectal adenocarcinoma and being fit to receive radiotherapy and/or chemotherapy by PVI. Randomisation by minimisation was used, stratified by cancer site (colon or rectum), age, timing of radiotherapy and surgeon. This method ensures balance between treatment groups for the specified factors (Pocock and Simon, 1975).

Information on age, sex and tumour size was collected at randomisation. Only data on modified Duke's stage were available, not tumour size.

A total of 3008 trial patients (82% of those originally randomised) who were resident in England and Wales were initially considered for analysis. We simplified follow-up by excluding patients resident in Scotland or Northern Ireland (257), as the Office for National Statistics (ONS) does not monitor their vital status, and those who emigrated after diagnosis (25). A further 204 patients were excluded as no ONS record could be found, and 41 because of missing dates or postcode, or because the cancer record had been cancelled. Postcode was required to assign a deprivation category, and the dates of birth, diagnosis (randomisation) and death are required to estimate survival by age. We analysed data for 2481 patients: 1326 with colon cancer and 1155 with rectal cancer (Table 1).

The AXIS trial patients were flagged on the National Health Service Central Register to provide follow-up information on vital status. The Office for National Statistics used the postcode of residence at diagnosis to assign each patient a deprivation score on the basis of the small area (electoral ward). For patients in England, we used the ward Carstairs score (Carstairs, 1995) for patients randomised during 1986–1995, and the ward income domain score from the index of multiple deprivation (IMD) 2000 (Department of the Environment Transport and the Regions, 2000) for those randomised during 1996–1997. For patients in Wales, we used the equivalent Welsh index. Patients were grouped into five categories, from least deprived to most deprived, based on the quintiles of the national distribution of scores for each ward (Coleman *et al*, 2004). The Office for National Statistics supplied anonymised data, including the deprivation category but not the patient's name, address, postcode or deprivation score, or the ward from which it was derived.

### Statistical analyses

All patients were followed up for at least 5 years to the end of 2003, the last year for which complete information on vital status was available. Survival was calculated in years as the interval between the date of randomisation and the earliest of the date of death, 31 December 2003 and the last date of follow-up, divided by 365.25. Survival probabilities were estimated 3-monthly for the first 6 months, then at 1 year, annually up to 5 years and finally at 7 years, using a maximum likelihood approach for individual data (Estève *et al*, 1990). Relative survival was estimated by deprivation category both for all patients and within each of the two 5-FU trial arms. We report relative survival at 1 and 5 years.

Relative survival (Ederer *et al*, 1961) estimates the probability of survival from the cancer by splitting the observed probability of death into two components: the cancer-related and the background probabilities of death. The background risk of death varies widely by age, sex and socioeconomic group; we used deprivation-specific life tables of all-cause mortality by single year of age at death, sex and calendar period (Cancer Research UK Cancer Survival Group, 2004). These were derived from the number of deaths in three successive calendar years, to provide a more robust estimation of national death rates at each single year of age. The 1990–1992 life tables were used to represent background mortality during 1986–95, and the 1997–1999 life tables for 1996–2001.

The ‘deprivation gap’ in relative survival in the trial was estimated with variance-weighted linear regression as the fitted difference between the ‘most affluent’ and ‘most deprived’ categories (Grizzle *et al*, 1969), and compared with the deprivation gap for the general population of England and Wales (Coleman *et al*, 2004). The gap is defined as negative if survival is lower in the ‘most deprived’ group.

### Missing data

Tumour stage was the only variable with missing values (239 patients, 10%). Multiple imputation (Rubin, 1987; Schafer, 1997) was used to account for this incompleteness. In this approach, a model is posited for the association between missing values and recorded values, using records in which stage data are available. This model is used to generate several replicate ‘completed’ data sets, containing imputed values in place of the missing values. Estimates of the parameters of interest in each completed data set, with their variance, are then pooled using multiple imputation rules (Rubin, 1987). Ten imputations were performed: simulation studies have shown no extra benefit from using more imputations for variables with up to 50% missing values (Little and Rubin, 1987; Rubin, 1996).

### Multivariable modelling using fractional polynomials

Multivariable regression using a generalised linear model with Poisson error (Dickman *et al*, 2004) was used to estimate the excess hazard of death of the trial patients over the background mortality. We also estimated the excess hazard ratio (reference: most affluent category) associated with deprivation, adjusting for the confounding effects of age at randomisation, sex, cancer site (colon, rectum) and stage. The fractional polynomials approach (Sauerbrei *et al*, 1999) was applied to each of the ten ‘completed’ data sets, to model potentially non-linear relationships of both the baseline excess hazard and the excess hazard ratio of the continuous prognostic factors. The variables time since randomisation, stage, sex and cancer site were forced into the model to adjust for the excess mortality hazard of deprivation. The interactions between follow-up time and deprivation or age were tested. The Akaike Information Criterion (AIC) was used to measure the goodness of fit of each model. All analyses were carried out using *Stata* algorithms (StataCorp, 1997; Dickman, 2006).

## Results

The 2481 patients were more or less equally distributed across the five deprivation categories in each of the main arms of the trial (data not shown) and in both arms combined (Table 1). Over 90% of patients were aged 40–79 years, 60.7% were male, and some 70% were diagnosed at Duke's stage B or C. Data on stage were missing for 9.6% of cases.

Across the five deprivation groups, relative survival ranged from 85.5 to 90.8% at 1 year after diagnosis and from 63.4 to 67.3% at 5 years (Table 2), but there was no evidence of a linear trend across deprivation groups. As reported in the original trial, survival at 1 and 5 years was similar in both arms of the trial (5-FU or not; data not shown).

**Figure 2:**
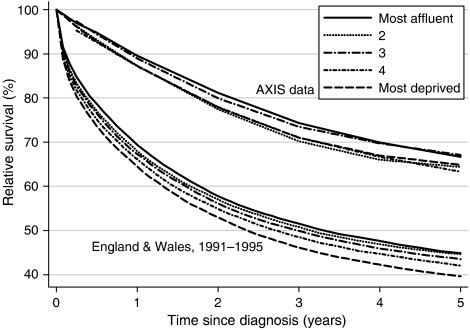
Relative survival (%) at 1 and 5 years, by deprivation category, in the AXIS trial (1989–97) and the general population of England and Wales (1991–1995).

For all patients combined, the fitted difference in survival between the most affluent and most deprived groups was −3.2% 1 year after diagnosis and −1.7% after 5 years; neither result was statistically significant (Table 2, Figure 2). The deprivation gap in survival at 1 and 5 years was smaller than that estimated in the general population of England and Wales for the calendar period 1991–1995, during which 80% of the trial patients were diagnosed. Results were similar when each trial arm was examined separately (data not shown).

The final model of the excess hazard ratio, after imputation of missing values for stage, included age, stage and site, as well as time since randomisation and deprivation category. The excess hazards for age and deprivation remained proportionate by time since randomisation.

The excess hazard of death within 5 years after diagnosis was between 9 and 20% higher in more deprived categories than in the most affluent group, after adjustment for time since randomisation, age, sex, cancer site and stage. The effect was not linear, and the overall effect of deprivation on the excess hazard of death was not statistically significant (Table 3). The excess hazard of death increased with tumour stage and, to a lesser extent, with age at randomisation. Rectal cancer patients and men both experienced a higher excess hazard of death.

## Discussion

Population studies have shown that the survival of colorectal cancer patients varies by deprivation category. By contrast, there was no indication of a deprivation gap in survival in this large-scale trial, either at 1 or 5 years after randomisation. There was no evidence of a deprivation gradient in the excess hazard of death after adjustment for time since randomisation, age, sex, tumour site and stage.

The AXIS study is one of the largest randomised trials of treatment for colorectal cancer (3681 patients). Detailed information on stage at diagnosis and treatment was available for 2481 patients who were randomised and could be followed up for at least 5 years in England and Wales. Random allocation and adherence to protocol ensured that all patients in a given arm followed similar treatment policies, regardless of socioeconomic status. Indeed, socioeconomic status as defined for this study was not known to the AXIS investigators or clinicians at the time: it was only derived after the trial, from the postcode of residence at diagnosis.

One- and 5-year survivals were much higher (13–25%) than among colorectal cancer patients in England and Wales in the same period (see Table 2) (Coleman *et al*, 1999). At first sight, this supports the view that cancer patients taking part in clinical trials get better treatment and have higher survival than the average population of cancer patients (Lara *et al*, 2001), but patients in the AXIS trial were younger than colorectal cancer patients in general. Higher survival was also expected, as most trial patients had early-stage, resectable disease. More important, there was no evidence of a deprivation gap in survival in the AXIS trial. This contrasts markedly with the significant deprivation gap in colorectal cancer survival at 1 and 5 years among patients diagnosed in England and Wales during 1991–1995.

If bias in selection for treatment could be ruled out, the results of this study suggest that the origin of the deprivation gradient in survival in the general population lies either in later diagnosis among more deprived patients or in socioeconomic differences in access to optimal treatment.

The socioeconomic gradients in 1- and 5-year survival in the AXIS study were small and not statistically significant. The survival gradients lie outside the 95% confidence intervals around the corresponding estimates for the general population of England and Wales (Coleman *et al*, 1999, 2001). Even in this large study, however, statistical power was limited, given the relatively small number of deaths in each deprivation group. We imputed missing values for tumour stage to minimise loss of power.

Stage at diagnosis and tumour site (colon or rectum) are both highly significant prognostic factors in colorectal cancer. The results confirm this, but adjustment for these factors had little impact on the excess hazard ratio in each socioeconomic group. Data from both arms of the trial were merged: this was justified because neither treatment had an impact on the excess hazard of death, which corroborates the main result of the AXIS trial (The AXIS collaborators, 2003).

The absence of a deprivation gradient in survival within this clinical trial suggests that lower survival among deprived patients in the general population may be due to health-care factors, such as delay in diagnosis, inequality in the thoroughness of diagnostic investigation or unequal access to optimal treatment. It could be argued that fewer patients were included from deprived groups than from affluent groups on the grounds of severe comorbidity that might have prevented treatment or led to the interruption of treatment because of adverse effects. Such differences might explain lower survival among deprived patients in the general population. In the AXIS trial, however, the socioeconomic distribution of patients was unknown at randomisation. The percentage of patients treated in the most deprived group in the trial was slightly lower than the comparable group of colorectal cancer patients in the general population, but the socioeconomic distribution of AXIS trial patients was still weighted toward the more deprived groups. These points argue against selection bias in the AXIS trial arising from preferential recruitment of more affluent patients on the grounds of lower comorbidity.

In the USA, survival was similar among blacks and whites in trials of adjuvant therapy for colon cancer (Dignam *et al*, 1999). One commentator remarked that race in the USA is a surrogate for socioeconomic status, adding, ‘Ultimately (this result) helps one understand that equal treatment yields equal outcome among patients with the same stage of disease, regardless of race’ (Brawley and Freeman, 1999). In similar vein, the findings from the AXIS study tend to suggest that equal treatment does yield equal outcome, regardless of socioeconomic status.

We can conclude that, given equal treatment at a given stage of disease, survival from colorectal cancer does not depend on socioeconomic status. This supports the notion that health-care system factors do underpin inequalities in survival in the general population. However, our study cannot determine directly whether inequalities in survival are due to differences in access to optimal treatment. We plan similar analyses of patients randomised in four other large trials, two of ovarian cancer and two of testicular cancer.

## Figures and Tables

**Table 1:** Number (%) of colorectal cancer patients by deprivation group

	**Deprivation category**											
	**Most affluent**		**2**		**3**		**4**		**Most deprived**		**No of patients**	
**Deprivation**	**No.**	**%**	**No.**	**%**	**No.**	**%**	**No.**	**%**	**No.**	**%**	**No.**	**%**
*(a) AXIS trial (1989–1997)*												
No. of patients	423	17.1	567	22.9	481	19.4	510	20.6	500	20.2	2481	100.0
												
*Site of tumour*												
Colon	234	17.7	327	24.7	259	19.5	258	19.5	248	18.7	1326	53.5
Rectum	189	16.4	240	20.8	222	19.2	252	21.8	252	21.8	1155	46.6
												
*Sex*												
Male	238	15.8	353	23.5	296	19.7	319	21.2	299	19.9	1505	60.7
Female	185	19.0	214	21.9	185	19.0	191	19.6	201	20.6	976	39.3
												
*Age (years)*												
Less than 40	8	24.2	10	30.3	4	12.1	6	18.2	5	15.2	33	1.3
40–59	81	15.3	135	25.6	106	20.1	109	20.6	97	18.4	528	21.3
60–79	304	17.4	376	21.6	338	19.4	357	20.5	369	21.2	1744	70.3
80 and over	30	17.1	46	26.1	33	18.8	38	21.6	29	16.5	176	7.1
												
*5-FU*												
No	217	18.0	279	23.1	223	18.5	240	19.9	249	20.6	1208	48.7
Yes	195	16.1	277	22.8	248	20.5	257	21.2	236	19.5	1213	48.9
Not randomised	11	18.3	11	18.3	10	16.7	13	21.7	15	25.0	60	2.4
												
*Duke's stage*												
A	56	19.2	68	23.3	59	20.2	59	20.2	50	17.1	292	11.8
B	171	17.5	226	23.2	201	20.6	194	19.9	183	18.8	975	39.3
C	117	15.2	181	23.5	137	17.8	165	21.5	169	22.0	769	31.0
D	44	21.4	44	21.4	37	18.0	45	21.8	36	17.5	206	8.3
Missing	35	14.6	48	20.1	47	19.7	47	19.7	62	25.9	239	9.6
												
*(b) General population of England and Wales (1991–1995)*												
No. of patients	17 800	14.3	21 438	17.3	24 826	20.0	28 954	23.3	31 229	25.1	124 247	100.0
												
*Site of tumour*												
Colon	11 222	14.7	13 555	17.7	15 557	20.3	17 650	23.0	18 607	24.3	76 591	61.6
Rectum	6578	13.8	7883	16.5	9269	19.5	11 304	23.7	12 622	26.5	47 656	38.4
												
*Sex*												
Male	9301	14.4	10 951	17.0	12 792	19.8	15 003	23.2	16 531	25.6	64 578	52.0
Female	8499	14.2	10 487	17.6	12 034	20.2	13 951	23.4	14 698	24.6	59 669	48.0
												
*Age (years)*												
Less than 40	219	15.3	208	14.6	256	17.9	350	24.5	397	27.8	1430	1.2
40–59	3229	16.5	3430	17.5	3802	19.4	4368	22.3	4739	24.2	19 568	15.8
60–79	10 245	13.9	12 584	17.0	14 679	19.9	17 205	23.3	19 159	25.9	73 872	59.5
80 and over	4107	14.0	5216	17.8	6089	20.7	7031	23.9	6934	23.6	29 377	23.6

**Table 2:** Relative survival (%) by deprivation category and deprivation gap (%) at 1 and 5 years: AXIS study population (diagnosed 1989–1997) and general population of England and Wales (1991–1995)

	**Study population**			**General population**	
				**Colon**	**Rectum**
	**Patients**	**Deaths**	**Relative survival**	**Relative survival**	
*1-year survival*					
Most affluent	423	52	90.8	67.2	73.2
2	567	98	85.5	65.4	72.5
3	481	71	88.6	65.1	71.0
4	510	85	87.2	63.6	69.8
Most deprived	500	86	86.0	61.6	68.8
Deprivation gap (%)			3.2	−5.3^a^	−4.6^a^
					
*5-year survival*					
Most affluent	423	186	66.7	45.0	45.0
2	567	251	64.5	44.3	45.3
3	481	205	67.3	43.2	44.0
4	510	241	63.4	42.1	41.7
Most deprived	500	238	65.0	39.6	39.4
Deprivation gap (%)			−1.7	−5.3^a^	−6.3^a^

a Statistically significant at 1%.

**Table 3:** Adjusted^a^ excess hazard ratios (EHR) of death within 5 years of diagnosis, with 95% confidence intervals (CI): colorectal cancer patients in AXIS trial

	**EHR**	**95% CI**	***P*-value**
*Deprivation*			
Most affluent	1.00		0.54
2	1.17	(0.92–1.51)	
3	1.09	(0.84–1.42)	
4	1.18	(0.91–1.52)	
Most deprived	1.20	(0.92–1.57)	
			
*Age at randomisation*			
10-year difference	1.07	(1.05–1.10)	0.06
			
*Duke's stage*			
A	1.00		<0.001
B	4.57	(1.82–11.46)	
C	14.36	(5.76–35.78)	
D	65.83	(26.11–165.96)	
			
*Tumour site*			
Colon	1.00		<0.001
Rectum	1.33	(1.14–1.55)	
			
*Sex*			
Male	1.00		0.10
Female	0.89	(0.76–1.06)	

a Adjusted for all variables in the table, and time since randomisation. Models fitted after multiple imputation of stage (see text).
